# Patterns of Substance Use Across the First Year of College and Associated Risk Factors

**DOI:** 10.3389/fpsyt.2015.00152

**Published:** 2015-10-27

**Authors:** Seung Bin Cho, Danielle C. Llaneza, Amy E. Adkins, Megan Cooke, Kenneth S. Kendler, Shaunna L. Clark, Danielle M. Dick

**Affiliations:** ^1^Department of African American Studies, Virginia Commonwealth University, Richmond, VA, USA; ^2^Department of Psychology, Virginia Commonwealth University, Richmond, VA, USA; ^3^Department of Psychiatry, Virginia Institute for Psychiatric and Behavioral Genetics, Virginia Commonwealth University, Richmond, VA, USA; ^4^Department of Pharmacotherapy and Outcome Science, Virginia Commonwealth University, Richmond, VA, USA; ^5^Department of Human and Molecular Genetics, Virginia Commonwealth University, Richmond, VA, USA

**Keywords:** substance use, risk/protective factors, latent transition analysis, college students, early adulthood, spit for science

## Abstract

Starting college is a major life transition. This study aims to characterize patterns of substance use across a variety of substances across the first year of college and identify associated factors. We used data from the first cohort (*N* = 2056, 1240 females) of the “Spit for Science” sample, a study of incoming freshmen at a large urban university. Latent transition analysis was applied to alcohol, tobacco, cannabis, and other illicit drug uses measured at the beginning of the fall semester and midway through the spring semester. Covariates across multiple domains – including personality, drinking motivations and expectancy, high school delinquency, peer deviance, stressful events, and symptoms of depression and anxiety – were included to predict the patterns of substance use and transitions between patterns across the first year. At both the fall and spring semesters, we identified three subgroups of participants with patterns of substance use characterized as: (1) use of all four substances; (2) alcohol, tobacco, and cannabis use; and (3) overall low substance use. Patterns of substance use were highly stable across the first year of college: most students maintained their class membership from fall to spring, with just 7% of participants in the initial low substance users transitioning to spring alcohol, tobacco, and cannabis users. Most of the included covariates were predictive of the initial pattern of use, but covariates related to experiences across the first year of college were more predictive of the transition from the low to alcohol, tobacco, and cannabis user groups. Our results suggest that while there is an overall increase in alcohol use across all students, college students largely maintain their patterns of substance use across the first year. Risk factors experienced during the first year may be effective targets for preventing increases in substance use.

## Introduction

During the transition from high school to the first year of college, critical changes in individual freedoms, responsibilities, and living conditions occur (1). The transition from adolescence to emerging adulthood is known to be associated with increased risk of substance use/abuse (1, 2), and college students have higher levels of substance use than their same age peers (3). Risky substance use among college students is widespread (4, 5), with 39% of students reporting binge drinking and 22% reporting illicit drug use (4). Substance use is associated with a number of adverse consequences in young adulthood, including academic problems, unwanted sexual encounters, legal consequences, injury, suicide, and death (6–8). Accordingly, it is important to understand the changes in substance use that occur across the critical first year when students go to college, and associated risk and protective factors. Using data from an incoming cohort of freshmen at a large diverse urban university we aimed to characterize patterns of substance use across the first year of entry to university.

One limitation of the existing literature on college student substance use is that it is focused largely on alcohol use (9–12). However, it is common for individuals to use more than one substance at the same time, particularly during drug experimentation phases in late adolescence and young adulthood (13–16). Importantly, patterns of comorbidity across substances show that different patterns of multiple substance use may have distinctive etiologies and consequences (10, 17–19). Thus, it is important to consider multiple substances simultaneously to better understand the etiology and consequences of substance use among college students. Further, the majority of comorbidity studies across adolescence and young adulthood have focused on alcohol, tobacco, and/or cannabis (9, 10, 17, 20, 21). Few studies have considered other forms of illicit drugs, despite evidence that the mean ages of initiating high-risk illicit drug use are clustered in early adulthood (22). In a cohort study of Swiss young men, there was evidence for distinctive subgroups of individuals characterized by the use of those high-risk illicit drugs in addition to other substances (23). Thus, incorporating measures of illicit drug use beyond marijuana use is important in understanding patterns of college student substance use. To address these gaps in the literature, we studied patterns of substance use that incorporated alcohol, nicotine, marijuana, and other drug use.

Identifying factors that influence substance use and abuse are important to prevent substance use-related negative consequences. Factors that influence substance use/abuse have been identified across a number of domains (24) such as personality (25, 26), cognitive (27–29), familial (30–32), and situational (2, 3). In addition, these factors may be differentially associated with substance use in males and females. For example, girls with low parental monitoring were more vulnerable to earlyonset drinking (33), and aggression has been shown to influence substance use more strongly in males (34). Most studies on risk factors of substance use have been designed to examine relationships between the factors and a single type of substance. However, given the co-occurrence of different types of substance use during early adulthood and the possibility of different etiological factors associated with different patterns of substance use, considering the effect of risk factors to overall patterns of multi substance use, rather than considering each substance separately, may be more informative to understanding the development of substance use among college students.

In the present study, we applied the latent transition analysis (LTA) (35) to repeatedly measured substance use from a cohort of first year college students, assessed upon entry into college and again midway through their spring semester, to identify patterns of substance use and associated factors. LTA is a longitudinal extension of latent categorical variable approaches (11, 36), which allows for identification of subgroups of individuals, called latent classes, based on multivariate patterns of responses. LTA is particularly suited to examine multiple substance use and change in patterns of use among college students, as it identifies subgroups of individuals based on the combined patterns of responses across different occasions. Specifically, LTA identifies groups of individuals at different time points based on their patterns of multiple responses (i.e., different substances), and estimates individuals’ changes in substance use patterns as transitions between the groups identified at each occasion. Covariates can be included in LTA as predictors of class memberships and transitions between classes.

Specifically, in this study, we used LTA to: (1) identify groups of individuals who share homogeneous patterns of alcohol, tobacco, cannabis, and other illicit drug use; (2) identify transitions between the groups across the first year of college; and (3) identify predictors of the group memberships and the transitions. To predict group memberships and transition patterns, variables from multiple domains, including personality traits, cognitive, situational, and familial factors, traumatic/stressful experiences, and internalizing symptoms (anxiety and depression), were included as covariates. A subset of the situational factors, traumatic/stressful experiences and internalizing symptoms, were measured during both fall and spring semesters. The variables measured at the spring semester indexed participants’ experiences during college. By incorporating multiple types of substance use, including both licit and illicit substances, by studying experiences across the first year of college in addition to pre-existing risk and protective factors, and by fitting models separately to data from males and females, this study represents, to our knowledge, the largest study of patterns of substance use across the transition to college.

## Materials and Methods

### Participants

The sample used in this study is part of the Spit for Science project, a university-wide research study at a large, public, urban university focused on understanding the development of substance use and emotional health outcomes in college students. Recruitment started in the fall semester of 2011. Invitations were sent to 3623 eligible freshman students who were 18 years or older at the time the survey was administered. Data collection protocols were approved by the Institutional Review Board. The authors assert that all procedures contributing to this work comply with the ethical standards of the relevant national and institutional committees on human experimentation and with the Helsinki Declaration of 1975, as revised in 2008. Study data were collected and managed using REDCap electronic data capture tools (37) hosted at Virginia Commonwealth University.

Among the invited students, 2056 (57%) completed the initial survey, and 1240 (60.3%) were females. The mean age of participants was 18.51 (SD = 0.45). Ethnicity profiles of the participants were representative of the broader population of the university: American Indian/Native Alaskan (*n* = 10, 0.5%), Asian (*n* = 311, 15.1%), Black/African American (*n* = 395, 19.2%), Hispanics/Latino (*n* = 120, 5.8%), Native Hawaiian/Pacific Islander (*n* = 17, 0.8%), White (*n* = 1056, 51.4%), and multiracial (*n* = 109. 5.3%). Participants were followed up during the spring semester of 2012, and 1562 students (76% of those who completed the initial survey) completed the follow-up survey. Additional details on the study can be found in a previously published introductory article (38).

### Measures

Responses measured at the initial fall and the follow-up spring surveys were used to identify the patterns of substance use.

#### Alcohol Use

*Alcohol use* was measured by the number of days of drinking during the last 30 days with a five-point scale: “never,” “monthly or less,” “two to four times a month,” “two to three times a week,” and “four or more times a week.”

#### Tobacco Use

*Tobacco use* was measured by three items assessing the number of days cigarettes, cigars, or hookah were used during the last 30 days, with five response categories: 0, 1–2, 3–11, 12–25, and 26–30 days per month. The responses across three items were combined into one tobacco use scale by taking the maximal use of any of the included tobacco products.

#### Cannabis Use

*Cannabis use* was measured by the number of instances of non-medical use using a three-point scale: “none,” “at least once,” and “six or more times.” Non-medical use was defined as use without a prescription, in greater amounts than prescribed, or for reasons other than recommended by a doctor. In the fall survey, the timeframe of response was the participant’s lifetime, but in the spring, it was limited to after starting college.

#### Illicit Drug Use

*Illicit drug use* in this study was limited to the use of sedatives, stimulants, cocaine, or opioids. The use of each drug was measured by the number of instances of non-medical use using a three-point scale: none, at least once, and six or more times. Timeframes of these items were the same as that of the cannabis use item. The responses were aggregated into a single variable by taking the maximal use of any type of illicit drug.

We included a wide array of potential predictors of class membership in our models. These covariates represent multiple domains (personality, cognitive, familial, individual, and situational) that have previously been associated with substance use in adolescent and young adult populations (2, 3, 24–32). The analyses were exploratory, aimed at examining whether any of these variables would differentially predict the patterns of multiple substance use that emerged from the LTA. The following variables were included as covariates of initial class memberships and transition patterns: personality subscales measured using the Big Five Inventory (39), impulsivity subscales using the UPPS (40), alcohol expectancies measured with the Brief Comprehensive Effects of Alcohol (41), a four-factor measure of drinking motives (28), peer deviance as measured by the proportion of friends committing deviant behaviors (42, 43), high school delinquency measured using items adapted from the Semi Structured Assessment of the Genetics of Alcoholism (44), general religiosity (45), number of potentially traumatic events from the Life Events Checklist (46), stressful events (47), parents’ parenting styles based on Parenting Styles Inventory (48), and internalizing symptoms based on Symptom Checklist 90 (49). All measures were selected to reflect previously used and validated scales with reasonable psychometric properties. More detailed information on the measures can be found in a previously published paper (38). Peer deviance, traumatic and stressful events, and internalizing symptoms were measured both at the initial and follow-up assessments. All other covariates were measured at the initial assessment.

### Statistical Analysis

We applied LTA to the substance use responses from the fall and spring semesters. LTA, in our study, identifies subgroups of individuals, called latent classes, with homogeneous patterns of substance use, separately for the fall and spring semesters, and change of use as transitions between the subgroups for the fall and spring. LTA estimates three primary sets of parameters. First, the prevalence of each latent class is estimated at the fall and spring semester, respectively. This set of parameters represents how large each subgroup is at each time point. Second, the item response probabilities estimate the representative pattern of substance use in each class. Item response probabilities define the characteristics of each class, and a label can be assigned based on item response probabilities. The last set of parameters is the transition probabilities. In this study, transition probabilities are the probabilities of being in a spring class given membership in a particular fall class. This set of parameters estimates how likely participants are to change their patterns of substance use across the first year of college.

We first applied latent class analyses to responses from the fall and spring separately to determine the number and properties of the classes to be used in the LTA. Selecting the optimal number of classes was generally guided by information criteria – Akaike Information Criteria (AIC) (50) and Bayesian Information Criteria (BIC) (51) – and likelihood ratio tests (LRTs) – Vuong-Lo-Mendel-Rubin (VLMR) (52) and Bootstrap likelihood ratio tests (BLRT) (53). Because information criteria penalize the complexity of models (i.e., number of parameters to be estimated), models with lower values of information criteria represent the balance between the model fit and parsimony and are preferred between competing models. LRTs test changes in the log likelihood between models with *k* and *k* − 1 class models. Insignificant *p*-values from LRTs indicate that a model with *k* classes fits no better than a model with *k* − 1 classes. We also considered the interpretability of item response profiles of identified classes in deciding the number of classes because response profiles of classes should be theoretically meaningful (11, 54). The LTA model was applied to the data from the fall and spring to estimate the probabilities of transitions between classes across the first year. The LTA model was specified based on the classes identified in the latent class analyses. Invariance of item response parameters between classes from the fall and spring was tested using χ^2^ differences between models with and without equality constraints on the parameters between fall and spring.

Finally, covariates were entered into the LTA model as predictors of the class memberships and transitions between classes across time. Each covariate was standardized and separately entered into the LTA model. In addition, separate LTA models were fit to data from men and women to detect potential differences in the effects of covariates. Personality and impulsivity subscales, alcohol expectancies, drinking motives, delinquency and peer deviance in high school, pre-college traumatic/stressful experiences, parenting style, and pre-college anxiety and depression symptoms were modeled to predict the initial class memberships as well as transitions between classes. Peer deviance, traumatic/stressful experiences, and depression and anxiety symptoms during the first year of college measured at the spring semester were modeled to predict only transitions between classes because the timeframe of these variables was subsequent to the initial measurement of substance use. We used Mplus version 7.1 (55) and its maximum likelihood estimator with robust standard errors (MLR) using a numerical integration algorithm for parameter estimations.

## Results

Table 1 provides descriptive statistics of the substance use measures from the fall and spring surveys. There was a notable overall increase of alcohol use (from 49.2 to 68.8% of use at least once a month). Overall use of other types of substances, except illicit drug use, also slightly increased, but not as much as the increase evidenced for alcohol.

**Table 1:** **Descriptive statistics of four substance uses in the fall and spring semesters**.

		Fall		Spring	
		Frequency	%	Frequency	%
Alcohol use	Never	884	50.8	470	31.2
	Monthly or less	210	12.1	418	27.8
	Two to four times a month	326	18.7	392	26.0
	Two to three times a week	243	14.0	198	13.2
	Four or more times a week	76	4.4	27	1.8
Tobacco	0 days per last 30 days	1197	59.8	911	60.3
	1–2 days per last 30 days	356	17.8	267	17.7
	3–11 days per last 30 days	253	12.6	159	10.5
	12–25 days per last 30 days	76	3.8	73	4.8
	26–30 days per last 30 days	119	5.9	100	6.6
Cannabis	None	1160	58.1	903	61.0
	At least once	247	12.4	215	14.5
	6+ times	590	29.5	362	24.5
Illicit drugs	None	1736	86.0	1239	81.8
	At least once, any	145	7.2	178	11.7
	6+ times, any	137	6.8	98	6.5

Table 2 summarizes the fit statistics of the latent class analyses from fall and spring semesters. Given that BIC performs generally better than AIC in selecting the correct number of classes (53), and, as described in the following sections, the interpretation of 3-class solution was straightforward, we retained three classes for both the fall and spring semesters in the following analyses. Item response profiles could not be constrained equal across fall and spring given that the difference in χ^2^ between the two models, with and without the equality constraints on the thresholds, was significant (Δχ^2^ = 421.198, Δdf = 36, *p* < 0.001). Thus, we fit LTA models that allowed different item response profiles for the fall and spring.

**Table 2:** **Fit statistics from LCA in the fall and spring**.

Fall number of classes	Number of parameters	Likelihood ratio	AIC	BIC	VLMR^a^	BLRT^b^
1	12	−7518.64	15061.29	15128.72		
2	25	−6684.92	13419.84	13560.33	0.0000	0.0000
3	38	−6590.99	13257.98	13471.53	0.0462	0.0000
4	51	−6555.75	13213.50	13500.10	0.7453	0.0000

**Spring number of classes**	**Number of parameters**	**Likelihood ratio**	**AIC**	**BIC**	**LRT**	**BLRT**

1	12	−6162.82	12349.65	12413.7		
2	25	−5609.59	11269.17	11402.7	0.0000	0.0000
3	38	−5516.20	11108.41	11311.4	0.0000	0.0000
4	51	−5500.81	11103.61	11376.0	1.0000	0.0652

*^a^*p*-values of Vuong-Lo-Mendell-Rubin likelihood ratio tests for *k* vs. *k* − 1 classes*.

*^b^*p*-values of Bootstrapped Likelihood ratio test for *k* vs. *k* − 1 classes*.

**Figure 1:**
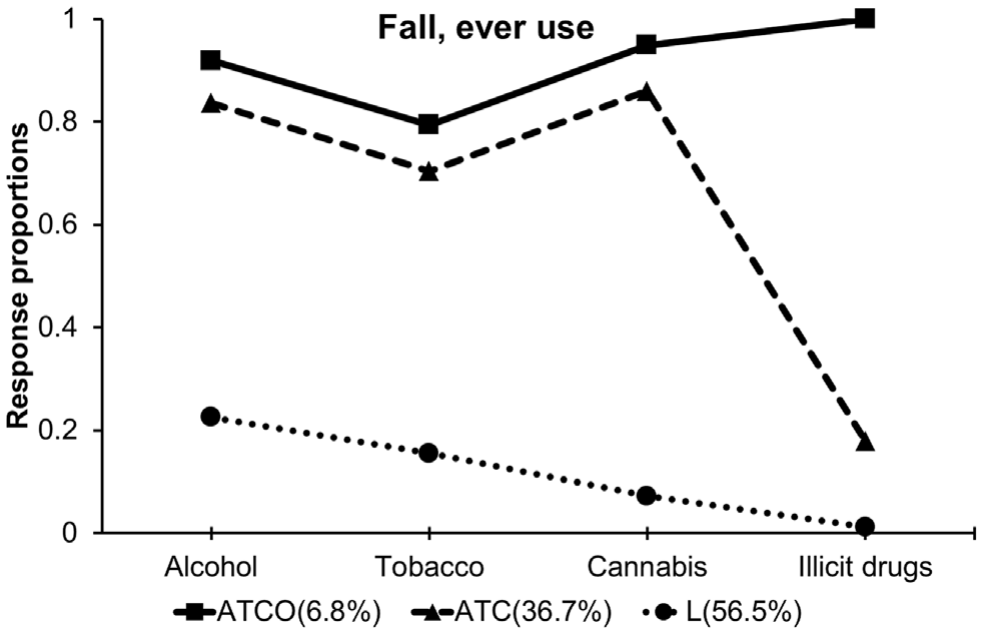
**Item response profiles from the initial fall classes showing the proportion of each class reporting ever use of each substance**.

**Figure 2:**
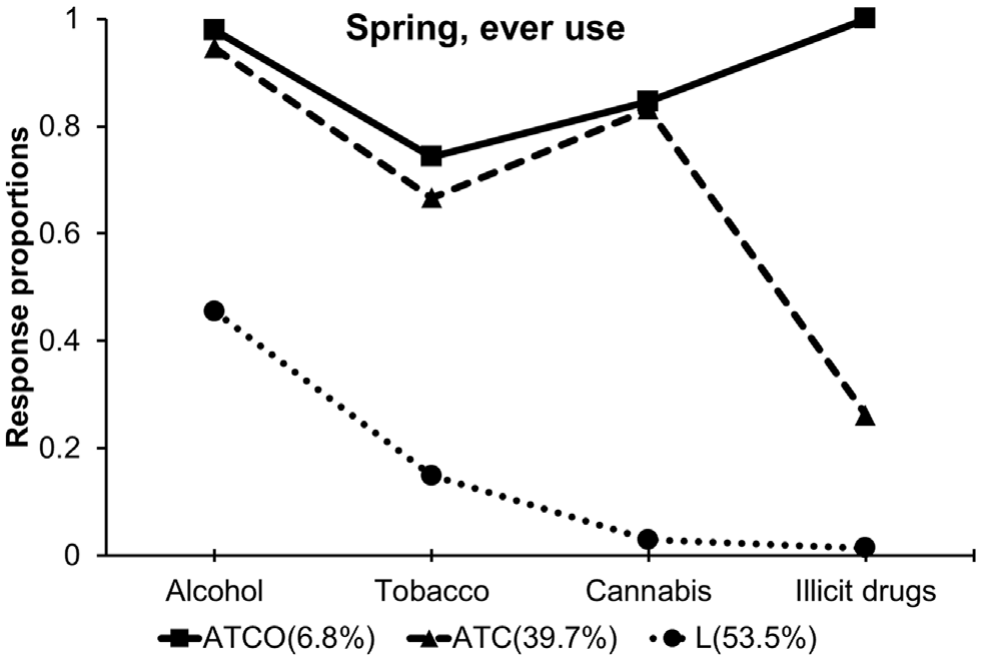
**Item response profiles from the spring classes showing the proportion of each class reporting ever use of each substance**.

Item response profiles and transition probabilities are provided in Table 3 and illustrated graphically Figures 1 and 2. For both fall and spring, the three classes represented individuals who used all substances, labeled ATCO, for “alcohol, tobacco, cannabis, other illicit drug use,” alcohol, tobacco, cannabis users, labeled ATC, and low substance users, labeled L, respectively, based upon relative differences in response profiles between classes. The L class was characterized by lower endorsement rates of all four substances compared to other classes. The ATC class was distinguished from the L class by elevated use of alcohol, tobacco and cannabis and by increased endorsement rate of “six or more times use” of cannabis. The ATCO class was characterized by higher levels of use across all four substances and was distinguished from the ATC class by increased endorsement of other illicit drug use. Some differences were observed in the response profiles between the fall and spring, since the response profiles were not constrained equal. Especially, overall alcohol use generally increased, while the proportions of extreme use (e.g., more than four times a week) decreased within equally labeled classes across the fall and spring semesters. Overall cannabis use decreased from fall to spring. Transition probabilities are summarized in the lower panel of Table 3. Most participants stayed in the same class across time; 2.6% of participants transitioned from the ATC to L class, whereas 7% of participants transitioned from the L to ATC class. This pattern of transition was labeled as the “Increasing” transition (Table 3).

**Table 3:** **Item response profiles and transition probabilities of three classes from LTA**.

		Fall			Spring		
	Classes	ATCO	ATC	L	ATCO	ATC	L
Proportions		6.8%	36.7%	56.5%	6.8%	39.7%	53.5%
**Alcohol**							
Never		0.081	0.163	0.775	0.021	0.054	0.545
Monthly or less		0.085	0.157	0.103	0.186	0.275	0.291
Two to four times a month		0.256	0.327	0.092	0.342	0.410	0.137
Two to three times a week		0.304	0.291	0.025	0.312	0.239	0.027
Greater than four times a week		0.274	0.063	0.004	0.140	0.022	0.000
**Tobacco**							
0 days		0.206	0.296	0.845	0.257	0.333	0.851
1–2 days		0.125	0.294	0.108	0.107	0.272	0.112
3–11 days		0.221	0.245	0.037	0.182	0.194	0.028
12–25 days		0.110	0.073	0.006	0.107	0.091	0.009
26–30 days		0.337	0.092	0.004	0.348	0.110	0.000
**Cannabis**							
None		0.051	0.140	0.928	0.154	0.168	0.971
At least once		0.045	0.243	0.057	0.393	0.447	0.024
Greater than 6 times		0.904	0.618	0.015	0.453	0.385	0.005
**Illicit drugs**							
None		0.000	0.821	0.988	0.000	0.738	0.986
At least once		0.000	0.179	0.012	0.000	0.262	0.014
Greater than six times		1.000	0.000	0.000	1.000	0.000	0.000

**Transition probabilities^a^**				**Spring classes**		
				**ATCO**	**ATC**	**L**

	**Fall classes**	ATCO		1.000^a^	0.000	0.000
		ATC		0.000	0.974	0.026	
		L		0.000	0.070	0.930	

*^a^Transition probabilities are the probabilities of transitions to one of the spring classes given the fall class, so the probabilities in the same row sum to 1*.

Each covariate was standardized and separately entered into the LTA, and separate models were fit to male and female participants to detect differential effects of covariates by sex. The effects of covariates on initial class memberships are shown in odds ratio scales, using the L class as the reference class (Tables 4–6). Covariates with odds ratios >1 can be regarded as risk factors, while odds ratios <1 can be interpreted as protective factors, because an increase in the risk or protective factor was associated with increased or decreased odds, respectively, of being classified into ATCO or ATC class membership, rather than the L class. The transition probability from the ATC to L class was too small (2.6%, Table 3) to yield reliable estimates of covariate effects, so we only focused on the increasing transition (L to ATC) using stable membership in the L class for the reference pattern of transition. Covariates with odds ratios >1 can be interpreted as risk factors for the increasing transition, because increase in the covariate is associated with higher odds of transitioning from the L to ATC class, compared to staying in the L class. The effects of covariates measured at the spring semester on the increasing transition were provided in the right panel of Table 6.

**Table 4:** **Effects of trait variables on initial class memberships and on the increasing transition**.

	Sex	Initial classes at fall				Increasing transition	
		ATCO		ATC		L → ATC	
		OR^a^ (95% CI)	*p*	OR (95% CI)	*p*	OR^b^ (95% CI)	*p*
**Personality**							
Extraversion	M	.	.	1.33 (1.11, 1.59)	0.002	.	.
	F	1.41 (1.12, 1.77)	0.004	1.36 (1.18, 1.57)	<0.001	.	.
Agreeableness	M	0.60 (0.47, 0.77)	<0.001	.	.	.	.
	F	0.67 (0.54, 0.83)	<0.001	.	.	.	.
Conscientiousness	M	0.52 (0.40, 0.69)	<0.001	0.73 (0.62, 0.86)	<0.001	.	.
	F	0.48 (0.38, 0.61)	<0.001	0.76 (0.66, 0.87)	<0.001	.	.
Neuroticism	M	1.72 (1.30, 2.28)	<0.001	.	.	.	.
	F	1.54 (1.18, 2.01)	0.002	.	.	.	.
Openness	M	1.90 (1.33, 2.72)	<0.001	1.47 (1.24, 1.75)	<0.001	.	.
	F	.	.	.	.	.	.
**Impulsivity**							
Negative	M	1.83 (1.32, 2.54)	<0.001	.	.	.	.
Urgency	F	1.97 (1.47, 2.65)	<0.001	.	.	.	.
Positive	M	1.61 (1.18, 2.21)	0.003	.	.	.	.
Urgency	F	1.61 (1.25, 2.08)	<0.001	.	.	.	.
Lack of premeditation	M	1.43 (1.08, 1.89)	0.012	1.27 (1.02, 1.59)	0.031	.	.
	F	1.96 (1.52, 2.53)	<0.001	1.33 (1.13, 1.56)	0.001	.	.
Lack of perseverance	M	.	.	.	.	.	.
	F	1.57 (1.22, 2.01)	<0.001	.	.	.	.
Sensation	M	2.03 (1.34, 3.08)	0.001	1.57 (1.22, 2.02)	<0.001	.	.
Seeking	F	1.66 (1.19, 2.32)	0.003	1.43 (1.23, 1.67)	<0.001	1.7 (1.06, 2.74)	0.028

*^a^Odds ratios used the L class as a reference class*.

*^b^Odds ratios used the transition pattern of staying in the L class as a reference transition pattern*.

**Table 5:** **Effects of alcohol expectancies and drinking motives on initial class memberships and the increasing transition**.

	Sex	Initial classes at fall				Increasing transition	
		ATCO		ATC		L → ATC	
		OR^a^ (95% CI)	*p*	OR (95% CI)	*p*	OR^b^ (95% CI)	*p*
**Alcohol expectancies**							
Sexuality	M	2.17 (1.61, 2.92)	<0.001	1.71 (1.41, 2.07)	<0.001	4.49 (1.49, 13.52)	0.008
	F	1.93 (1.51, 2.47)	<0.001	1.61 (1.39, 1.86)	<0.001	.	.
Impairment	M	.	.	0.78 (0.64, 0.96)	0.017	.	.
	F	0.80 (0.65, 0.99)	0.037	0.85 (0.74, 0.98)	0.022	.	.
Risk/aggression	M	.	.	0.76 (0.64, 0.90)	0.002	.	.
	F	.	.	.	.	.	.
Tension reduction	M	1.93 (1.50, 2.47)	<0.001	1.99 (1.63, 2.43)	<0.001	.	.
	F	1.43 (1.12, 1.83)	0.004	1.34 (1.17, 1.53)	<0.001	2.79 (1.7, 4.58)	<0.001
Liquid courage	M	1.84 (1.39, 2.45)	<0.001	1.47 (1.23, 1.75)	<0.001	.	.
	F	1.50 (1.13, 2.00)	0.005	1.34 (1.16, 1.54)	<0.001	.	.
Self-perception	M	0.59 (0.43, 0.79)	0.001	0.48 (0.39, 0.58)	<0.001	.	.
	F	0.44 (0.33, 0.60)	<0.001	0.46 (0.40, 0.54)	<0.001	0.57 (0.36, 0.93)	0.023
Sociability	M	2.93 (1.88, 4.57)	<0.001	2.39 (1.84, 3.11)	<0.001	.	.
	F	2.29 (1.46, 3.58)	<0.001	2.59 (1.99, 3.36)	<0.001	.	.
**Drinking motives**							
Sociability	M	2.96 (1.86, 4.70)	<0.001	3.10 (2.28, 4.22)	<0.001	.	.
	F	2.61 (1.80, 3.80)	<0.001	2.34 (1.93, 2.84)	<0.001	.	.
Coping	M	1.71 (1.28, 2.27)	<0.001	1.30 (1.03, 1.64)	0.030	.	.
	F	2.03 (1.58, 2.61)	<0.001	1.60 (1.34, 1.91)	<0.001	.	.
Enhancement	M	4.20 (2.77, 6.38)	<0.001	3.95 (2.85, 5.46)	<0.001	.	.
	F	4.64 (3.01, 7.16)	<0.001	3.00 (2.35, 3.82)	<0.001	.	.

*^a^Odds ratios used the L class as a reference class*.

*^b^Odds ratios used the transition pattern of staying in the L class as a reference transition pattern*.

**Table 6:** **Effects of covariates measured in the fall on initial class memberships and the increasing transition and effects of covariates measured at spring on the increasing transition**.

	Sex	Initial classes at fall				Increasing transition	
		ATCO		ATC		L → ATC	
		OR^a^ (95% CI)	*p*	OR (95% CI)	*p*	OR^b^ (95% CI)	*p*
Sex (female)		.	.	0.64 (0.54, 0.77)	<0.001	.	.
Delinquency – high school	M	4.76 (3.21, 7.06)	<0.001	3.08 (2.2, 4.33)	<0.001	.	.
	F	5.25 (3.92, 7.05)	<0.001	2.97 (2.4, 3.68)	<0.001	1.48 (1.03, 2.11)	0.032
Religiosity	M	0.54 (0.40, 0.73)	<0.001	0.73 (0.62, 0.87)	<0.001	.	.
	F	0.63 (0.51, 0.79)	<0.001	0.71 (0.62, 0.81)	<0.001	.	.
Parenting – involvement	M	.	.	.	.	.	.
	F	0.77 (0.62, 0.95)	0.015	.	.	.	.
Peer deviance – high school	M	13.0 (7.8, 21.8)	<0.001	4.14 (3.11, 5.50)	<0.001	3.01 (1.68, 5.38)	<0.001
	F	12.6 (8.5, 18.6)	<0.001	4.35 (3.32, 5.72)	<0.001	.	.
Peer deviance – in college	M	.	.	.	.	1.63 (1.20, 2.20)	0.002
	F	.	.	.	.	4.89 (2.37, 10.1)	<0.001
Traumatic events – before college	M	1.30 (1.01, 1.66)	0.040	1.20 (1.01, 1.42)	0.035	.	.
	F	1.74 (1.37, 2.22)	<0.001	1.36 (1.18, 1.56)	<0.001	.	.
Traumatic events – in college	M	.	.	.	.	.	.
	F	.	.	.	.	2.37 (1.64, 3.42)	<0.001
Stressful events – before college	M	1.48 (1.15, 1.9)	0.002	1.34 (1.11, 1.63)	0.003	.	.
	F	1.94 (1.57, 2.4)	<0.001	1.69 (1.47, 1.95)	<0.001	.	.
Stressful events – in college	M	.	.	.	.	3.17 (1.50, 6.71)	0.003
	F	.	.	.	.	2.47 (1.81, 3.37)	<0.001
Anxiety – before college	M	1.68 (1.37, 2.05)	<0.001	.	.	.	.
	F	1.58 (1.31, 1.90)	<0.001	1.14 (0.99, 1.31)	0.060	.	.
Anxiety – in college	M	.	.	.	.	1.96 (1.01, 3.80)	0.046
	F	.	.	.	.	.	.
Depression – before college	M	1.83 (1.46, 2.28)	<0.001	.	.	1.67 (1.02, 2.72)	0.040
	F	1.65 (1.36, 1.99)	<0.001	1.18 (1.04, 1.35)	0.014	.	.
Depression – in college	M	.	.	.	.	2.00 (1.15, 3.48)	0.014
	F	.	.	.	.	1.60 (1.06, 2.42)	0.025

*^a^Odds ratios used the L class as a reference class*.

*^b^Odds ratios used the transition pattern of staying in the L class as a reference transition pattern*.

Personality and impulsivity as covariates for the initial class memberships and transitions were summarized in Table 4. Among males, the personality subscales of extraversion, neuroticism, and openness were risk factors, while agreeableness and conscientiousness were protective factors. All impulsivity dimensions were risk factors, except for the lack of perseverance in males. Among females, extraversion and neuroticism functioned as risk factors, while agreeableness and conscientiousness functioned as protective factors of initial ATCO or ATC class membership. Sensation seeking was a risk factor of the increasing transition among females, as higher scores were associated with higher odds of transitioning.

The effects of cognitive factors – alcohol expectancies and drinking motives are reported in Table 5. Among males, expectancies of enhanced sexual pleasure (sexuality), feeling relaxed (tension reduction), feeling powerful and brave (liquid courage), and being more sociable (sociability) were risk factors. Further, the sexuality scale was predictive of the increasing transition. Protective factors among men were feeling dizzy and clumsy (cognitive impairment), acting risky and aggressive (risk/aggression), and feeling guilty and moody (self-perception). Among females, similar patterns were observed for the effects of alcohol expectancy scales on initial class memberships, except for the risk/aggression scale. Predictors of the increasing transition were expecting tension reduction and self-perception. Subscales of drinking motives – to be more sociable (sociability), to cope with difficulties (coping), and to enhance mood (enhancement) – functioned as risk factors among males and females. Drinking to conform to social norms (conformity) was not associated with either initial class memberships or the transition.

Covariates that measured the effects of pre-established conditions, prior to or at the start of college, as measured in the fall semester, are reported in Table 6. Peer deviance and delinquent behaviors in high school, traumatic and stressful experiences, and symptoms of anxiety and depression were risk factors for initial ATCO and ATC class memberships among males and females. Religiosity was a protective factor against initial ATCO and ATC class memberships. Being female was protective against initial ATC class, and an involved parenting style was protective against ATCO class membership only in females. High school delinquency in females, high school peer deviance in males, and depression in males were also risk factors of the increasing transition.

Table 6 also summarizes the effects of covariates measured in the spring semester on the increasing transition. These covariates represent participants’ experiences during the first year of college. All of these covariates – peer deviance, traumatic and stressful events, anxiety, and depression in college – were risk factors for the increasing transition. It is notable that among the same set of pre-college variables, only high school peer deviance and depression in males were predictive of the increasing transition. Sex differences were observed for traumatic events and anxiety symptoms, in that experiencing more traumatic events was a risk factor for the increasing transition only in females, and anxiety was a risk factor for the transition only in males.

## Discussion

This study represents one of the largest, most comprehensive studies of patterns of substance use across the first year of college, a key transitional period associated with new independence and life changes. We identified subgroups of individuals among first year college students with different levels of substance use based on four types of substances – alcohol, tobacco, cannabis, and other illicit drugs. We also examined risk and protective factors across multiple domains that are associated with initial class memberships and a transition between classes. The current study builds upon the extant literature on college students’ substance use in two primary ways. First, we included multiple types of substance use, including riskier types of illicit drugs – sedatives, stimulants, cocaine, and opioids. Most of the previous work on substance use across the transition to college has omitted such riskier types of substances or focused on alcohol use (56, 57). By including multiple drug categories in our LTA, we were able to differentiate a class characterized by the use of illicit drugs in addition to alcohol, tobacco, and cannabis, from a class characterized by the use of alcohol, tobacco, and cannabis only. Similar results were observed from a study of Swiss young men (23), which identified a class of alcohol, tobacco, and cannabis users and another class characterized by illicit drug use additional to the three substances. Identification of a distinct class of illicit drug users underscores the importance of including riskier types of drugs when studying patterns of comorbidity. Second, we included covariates that represented participants’ experiences in college: peer deviance, traumatic/stressful experiences, and symptoms of anxiety and depression during the first year of college. These covariates were especially predictive of the increasing transition to a higher level of substance use while the corresponding variables assessed in the fall semester were mostly not. Our results illustrate the importance of risk factors across a variety of domains on initial patterns of substance use upon entry to college, but the proximity of college related experiences as being particularly important for changes in patterns of use.

One of the most notable findings from this study is the high consistency in class membership from the fall to spring semesters. Based on the result of LTA, most participants in our sample stayed in the same class during the first year of college: 7% of participants transitioned from the L to ATC class, while only 2.6% of participants transitioned from the ATC to L class. These results suggest that freshmen students maintain substance use patterns that were present at the start of the first semester and challenge the idea that college acts as a teratogenic agent that causes students to take up risky substance use patterns. Immobility between classes has also been observed in previous applications of LTA to alcohol and other substances (12, 23, 58). In addition, in a previous study, pre-college heavy drinking was reported to be the strongest predictor of heavy drinking in the first semester, suggesting that high-risk users have previously established substance use patterns prior to starting college (56). This could have important implications for prevention and intervention of substance use on college campuses. We do note, however, that the substance that showed the most dynamic change during the first year of college was alcohol, with an increase in alcohol use evident across all classes.

Predictors of initial patterns of substance use were largely consistent with previous studies, showing the important roles of personality and cognitive and situational factors. Most predictors were associated with both initial ATC and ATCO classes. However, the urgency dimension of impulsivity and two personality dimensions – (decreased) agreeableness and neuroticism – were uniquely associated with the riskier ATCO class only. There were more similarities than differences in risk factors affecting males and females, consistent with previous studies [(59), for example]. However, a few sex differences are worth noting. Only in females, lack of perseverance was associated with the ATCO class, and parental involvement was protective against the ATCO class membership. Pre-college anxiety and depression were associated with the ATCO class in males only.

Only a small number of covariates that were predictive of the initial patterns of substance use were also predictive of the increasing transition, from the fall L class to spring ATC class. None of personality subscales were associated with the transition. Sensation seeking was the only impulsivity dimension associated with the increasing transition. Alcohol expectancy of enhanced sexual pleasure (sexuality) in males was one of the few cognitive factors associated with the increasing transition. Expecting tension reduction and feeling guilty or moody (self-perception) were associated with the transition in females. However, *all covariates reflecting experiences during the first year of college (peer deviance, traumatic/stressful experiences, anxiety, and depression) were associated with the increasing transition*. These findings suggest that pre-existing risk/protective factors are not as important as first-year situational and environmental factors in predicting changes of substance use during the first year of college. This result is broadly consistent with a previous LTA of substance use among college students (60) wherein transitions during the first year were not predicted by demographic and pre-college factors. Our findings emphasize the importance of college experiences and offer a focus for prevention efforts.

### Limitations

Although our study provides a parsimonious way to describe the substance use of first year college students and associated factors, the results should be interpreted within the context of several limitations. First, although the timeframe employed in our study was designed to study patterns of substance use across the first year of college as students commence this important life phase, following these students across time will allow us to examine the longer-term changes in substance use across the college years. Second, due to estimation of the effect of each covariate separately, joint effects of covariates, such as moderations and mediations between factors, were not examined. One of the main goals of our study was to broadly explore factors associated with substance use patterns and changes in an exploratory fashion by covering a wide domain of potential risk and protective factors, providing the basis for further focused studies on specific factors. Relevant covariates identified in our study can be a focus of future investigations. For example, an important finding of the present study is the effect of college experiences on the increasing transition of substance use. Investigating how these variables interact with pre-existing characteristics over a longer assessment period could provide valuable information for effective intervention efforts. We also note that our surveys did not explicitly ask about the use of novel psychoactive substances, which have grown in popularity and availability in recent years, particularly among young adults (61–63). This will be an important area for future study in college populations.

## Conclusion

In summary, our analyses expand on the extant literature on college student substance use in two important ways. First, we identified a subgroup of students with increased risk of substance use by incorporating riskier types of drugs into the analyses additional to alcohol, tobacco, and cannabis. Second, we highlighted the importance of students’ experience after starting college in developing substance use behavior by including college experience variables into the LTA model, as predictors of the increasing transition pattern. This result implies that students’ experience after college entrance may be an important target in reducing the development of risky substance use. Our analyses also have implications for prevention and intervention efforts. First, given that the ATCO class represents a potentially more problematic pattern of substance use, covariates that only affect this class (neuroticism and anxiety, for example) may warrant further attention. Secondly, the wide variety of risk domains that predicted initial patterns of substance use indicate that prevention programs should address a breadth of potential risk factors. Finally, the high stability of substance use patterns across the first year of college suggest that prevention programs should be targeted at individuals as they enter college (or even prior to entry), since high-risk patterns are evident from the start of college attendance.

## Conflict of Interest Statement

The authors declare that the research was conducted in the absence of any commercial or financial relationships that could be construed as a potential conflict of interest.
